# Using framework-based synthesis for conducting reviews of qualitative studies

**DOI:** 10.1186/1741-7015-9-39

**Published:** 2011-04-14

**Authors:** Mary Dixon-Woods

**Affiliations:** 1 Social Science Research Group, Department of Health Sciences, School of Medicine, University of Leicester, Leicester, UK

## Abstract

Framework analysis is a technique used for data analysis in primary qualitative research. Recent years have seen its being adapted to conduct syntheses of qualitative studies. Framework-based synthesis shows considerable promise in addressing applied policy questions. An innovation in the approach, known as 'best fit' framework synthesis, has been published in *BMC Medical Research Methodology *this month. It involves reviewers in choosing a conceptual model likely to be suitable for the question of the review, and using it as the basis of their initial coding framework. This framework is then modified in response to the evidence reported in the studies in the reviews, so that the final product is a revised framework that may include both modified factors and new factors that were not anticipated in the original model. 'Best fit' framework-based synthesis may be especially suitable in addressing urgent policy questions where the need for a more fully developed synthesis is balanced by the need for a quick answer.

Please see related article: http://www.biomedcentral.com/1471-2288/11/29.

## Introduction

As the appetite for more holistic overviews of research evidence has grown, the last 10-15 years have seen increasing interest and activity directed at developing methods for synthesising qualitative studies [1]. Some of these methods might be fairly described as 'new' techniques that have been developed specifically for the purpose of conducting synthesis, while others might more properly be seen as adaptations of approaches that were originally intended for primary research [2]. Framework analysis is one of the latter. Developed during the 1980s by the UK-based National Centre for Social Research, and explicitly oriented towards applied policy questions, framework analysis is a matrix-based method involving the construction of thematic categories into which data can be coded [3]. One important feature of the approach is that, unlike some other qualitative methods, it allows themes or concepts identified *a priori *to be specified as coding categories from the outset, and to be combined with other themes or concepts that emerge *de novo *by subjecting the data to inductive analysis. A practical benefit of doing this is that it enables questions or issues identified in advance by various stakeholders (such as policymakers, practitioners, or user groups) to be explicitly and systematically considered in the analysis, while also facilitating enough flexibility to detect and characterise issues that emerge from the data.

Framework analysis has become hugely popular as a way of conducting analysis of primary qualitative data, especially in areas of healthcare with policy relevance. A recent study, for example, used framework analysis in a study of mothers' interpretations of dietary recommendations [4]. Because the study had been guided by social learning theory, and because the researchers were interested in comparing dietary beliefs and behaviours across social classes, the ability of framework analysis to cope with categories specified in advance of the data collection made it a very appropriate choice of analytic strategy. A further advantage is the use of charting techniques, which help not only in enhancing the transparency of coding, but also with teamwork in relation to analysis. Many of the properties that make framework analysis an appealing option for those conducting primary research make 'framework-based synthesis' a potentially equally attractive option for those seeking to conduct a synthesis of studies. A new article by Carroll *et al *[5] published this month in *BMC Medical Research methodology *reports an interesting evolution of the framework-based synthesis approach.

## Discussion

Framework analysis has previously been adapted for use in synthesis of qualitative evidence by Oliver and colleagues [6], who developed a multidimensional framework for analysing public involvement in health services research. They employed an iterative process involving familiarization with the literature, gradually developing a conceptual framework based on concepts derived from the review question and the theoretical and empirical literature, applying the framework systematically to evidence from the studies included in the review, and constructing a chart for each key dimension with distilled summaries from all relevant documents. The charts were used to map the range and nature of public involvement and to find associations between themes. Several benefits of this approach were noted by Oliver *et al*., including widening the scope of the review to include relevant topics identified by lay people, and the creation of data displays that could be viewed and assessed by people other than the primary analyst. Other examples of the application of the framework approach to the conduct of synthesis have now begun to appear, though it is dismaying that these and other syntheses of qualitative evidence continue to label themselves with the confusing, tautological, and inappropriate term 'meta-synthesis' [7].

In the recent *BMC Medical Research Methodology *manuscript [5], Carroll and colleagues develop the framework-based synthesis approach, focusing on the views of adults taking chemopreventative agents (such as aspirin or vitamins) in an effort to prevent colorectal cancer. One of the novel features of their approach is their use of a conceptual model that was used as an initial starting point for coding the evidence from 20 studies. This conceptual model was chosen because of its broad applicability to the area under review, and the authors did not engage in the more lengthy process of model specification that is often more characteristic of framework synthesis. They augmented analysis using this prespecified model with analysis that was more inductive, and ended up generating a revised conceptual model that provided a 'best fit' to the evidence reported in the studies they reviewed. The revised model included some factors that were absent from the original model, as well as adjustments to factors that had been reported in that model.

Carroll and colleagues emphasise the advantages of this kind of approach when time is short and the demand for policy-relevant evidence is urgent. It enables focusing of the research on the priorities of those commissioning the work, while still leaving some room for finding the 'best fit' in the light of what the evidence actually reports. Of course, like framework analysis for primary research [8] there are downsides of the approach too. Reviewers who have made a hefty investment in an initial conceptual model may be unconsciously motivated to recover the sunk costs of that model, and as a consequence tend to neglect evidence that presents a fundamental challenge. Putting more time into specifying the model, using a wider range of literature, and gaining the views of a wider range of stakeholders may all be important in improving the legitimacy and validity of any ensuring synthesis. There are also the usual risks of framework analysis that it can tend to suppress interpretive creativity, and thus reduce some of the vividness of insight seen in the best qualitative research. Nonetheless, as Carroll and colleagues argue, framework-based synthesis using the 'best fit' strategy is, in the right hands, likely to be a highly pragmatic and useful approach for a range of policy urgent questions.

## Conclusions

Framework-based synthesis is an important advance in conducting reviews of qualitative synthesis. The 'best fit' strategy is a variant of this approach that may be very helpful when policymakers, practitioners or other decision makers need answers quickly, and are able to tolerate some ambiguity about whether the answer is the very best that could be given.

## Competing interests

The author declares that they have no competing interests.

## Pre-publication history

The pre-publication history for this paper can be accessed here:

http://www.biomedcentral.com/1741-7015/9/39/prepub
